# Non‐invasive, ratiometric determination of intracellular pH in *Pseudomonas* species using a novel genetically encoded indicator

**DOI:** 10.1111/1751-7915.13439

**Published:** 2019-06-04

**Authors:** Alejandro Arce‐Rodríguez, Daniel C. Volke, Sarina Bense, Susanne Häussler, Pablo I. Nikel

**Affiliations:** ^1^ Department of Molecular Bacteriology Helmholtz Centre for Infection Research 38124 Braunschweig Germany; ^2^ The Novo Nordisk Foundation Center for Biosustainability Technical University of Denmark 2800 Kongens Lyngby Denmark

## Abstract

The ability of *Pseudomonas* species to thrive in all major natural environments (i.e. terrestrial, freshwater and marine) is based on its exceptional capability to adapt to physicochemical changes. Thus, environmental bacteria have to tightly control the maintenance of numerous physiological traits across different conditions. The intracellular pH (pH_i_) homoeostasis is a particularly important feature, since the pH_i_ influences a large portion of the biochemical processes in the cell. Despite its importance, relatively few reliable, easy‐to‐implement tools have been designed for quantifying *in vivo* pH_i_ changes in Gram‐negative bacteria with minimal manipulations. Here we describe a convenient, non‐invasive protocol for the quantification of the pH_i_ in bacteria, which is based on the ratiometric fluorescent indicator protein PHP (**pH** indicator for ***P**seudomonas*). The DNA sequence encoding PHP was thoroughly adapted to guarantee optimal transcription and translation of the indicator in *Pseudomonas* species. Our PHP‐based quantification method demonstrated that pH_i_ is tightly regulated over a narrow range of pH values not only in *Pseudomonas*, but also in other Gram‐negative bacterial species such as *Escherichia coli*. The maintenance of the cytoplasmic pH homoeostasis *in vivo* could also be observed upon internal (e.g. redirection of glucose consumption pathways in *P*. *putida*) and external (e.g. antibiotic exposure in *P*. *aeruginosa*) perturbations, and the PHP indicator was also used to follow dynamic changes in the pH_i_ upon external pH shifts. In summary, our work describes a reliable method for measuring pH_i_ in *Pseudomonas*, allowing for the detailed investigation of bacterial pH_i_ homoeostasis and its regulation.

## Introduction

Microbial cells have a remarkable ability to maintain physiological homoeostasis under a wide variety of environmental conditions. Thereby, the tight control of the intracellular pH (pH_i_, i.e. cytoplasmic pH) is of particular importance (Slonczewski *et al*., 2009), because pH_i_ influences enzyme activity, protein stability, nucleic acid structure and many other biological processes (Booth, 1985). It is well known that few species of adapted microbes can grow under extreme pH conditions. Bacterial life is possible even at pH values close to 0 (Nordstrom *et al*., 2000) or above pH = 13 (Roadcap *et al*., 2006). Nonetheless, the cytoplasmic pH value is remarkably similar for most bacteria, even when exposed to extreme pH environments (Krulwich *et al*., 2011; Martínez *et al*., 2012). Generally, bacteria have evolved a series of mechanisms to maintain this tight regulation of pH_i_ homoeostasis. A major strategy to this effect is to regulate the activity of proton (H^+^) transporters, such as H^+^‐pumping respiratory chain complexes and H^+^‐coupled ATPases or cation/H^+^ antiporters (Krulwich *et al*., 2011). As H^+^ ions are transported either way across the cell membrane by these mechanisms, major changes in the transmembrane pH gradient (ΔpH) are also generated. Since both ΔpH and the transmembrane electrical potential (ΔΨ) are directly connected to the H^+^‐motive force (PMF; given that PMF = ΔΨ – 60 × ΔpH at 25°C), the demand of pH homoeostasis directly influences the overall energetic status of the cell (Deamer and Nichols, 1989).

Several methods have been developed to monitor the pH_i_ in bacteria (Slonczewski *et al*., 2009; Chen and Lo, 2016). Many of them rely on the measurement of *in vivo* ^31^P nuclear magnetic resonance (NMR) spectra (Roberts *et al*., 1981; Sijbesma *et al*., 1996) or on the ratiometric fluorescence of specific chemical dyes (Molenaar *et al*., 1991; Breeuwer *et al*., 1996; Riondet *et al*., 1997; Awaji *et al*., 2001; Hunter and Beveridge, 2005; Rupprecht *et al*., 2017). While NMR is a laborious and not widely accessible technique, some of the fluorescent chemical probes commercially available are usually expensive, short‐lived and their use for pH_i_ determination requires a number of time‐consuming manipulations. In addition to these drawbacks, some fluorescein‐based fluorophores can rapidly leak out of cells once they are liberated in the cytosol *via* intracellular hydrolysis (Han and Burgess, 2010). Other chemical dyes also pose problems due to their (i) low permeability (which in turn result in uneven loading of dyes within a cell population), (ii) high chemical instability and (iii) cytotoxicity. Derivatives of fluorescent proteins that respond to changes in pH, including the model green fluorescent protein (GFP), have been described in the past decades (Olsen *et al*., 2002; Mahon, 2011; Martínez *et al*., 2012; Rupprecht *et al*., 2017; Reifenrath and Boles, 2018). The expression of genes encoding fluorescent proteins and the detection of the resulting fluorescence signals are an inexpensive and fast technique, enabling online measurements *in vivo*, requiring considerably less technical effort than other techniques deployed for pH_i_ determination. The dependence of the fluorescence intensity on the pH_i_ in GFP derivatives is based on the direct protonation of the phenolate moiety of the fluorophore derived from tryptophan, an extremely fast exchange reaction (McAnaney *et al*., 2005). Thus, fluorescence determinations allow for the assessment of the rate of pH_i_ change in response to rapid shifts in extracellular pH. Additionally, the continuous expression of the protein allows for live‐imaging over a longer timescale than is possible using chemical fluorophores (Grillo‐Hill *et al*., 2014).

Given the importance of assessing cytoplasmic pH values to understand the principles governing metabolic homoeostasis and energy conservation mechanisms in bacteria, the improvement of non‐invasive methods for calculating the pH_i_ is needed. In the present work, we propose a standard, non‐invasive method for the determination of pH_i_ in *Pseudomonas* species, as well as other Gram‐negative bacteria such as *Escherichia coli*. This procedure is based on the enhanced expression of a pH‐sensitive variant of GFP, and the ratiometric assessment of pH_i_ against cells equilibrated to a range of physiologically relevant pH values. The usability of this novel tool for the quantification of pH_i_ in Gram‐negative bacteria is illustrated by investigating the changes in pH homoeostasis brought about by internal metabolic perturbations in the cell factory platform *Pseudomonas putida* KT2440 and by antibiotic treatment in the opportunistic pathogen *P*. *aeruginosa*. The PHP indicator was also used to explore the response of pH_i_ in *E*. *coli* upon several perturbations. Moreover, the intrinsic compatibility of our tool with plasmids following the *Standard European Vector Architecture* (SEVA) (Silva‐Rocha *et al*., 2013) allows to modulate the replication and expression of *PHP* in other bacteria. As indicated by the examples below, the PHP indicator constitutes a flexible, non‐invasive and easy‐to‐implement tool to study both endogenous and exogenous pH perturbations in bacteria.

## Results and discussion

### Construction, benchmarking and calibration of the PHP indicator as a reliable tool to calculate the cytoplasmic pH in *Pseudomonas*

Our earlier attempts to use previously characterized ratiometric fluorescent proteins as pH_i_ indicators in *Pseudomonas* species (and, in particular, *P*. *putida* and *P*. *aeruginosa*) did not yield satisfactory signal‐to‐noise ratios. We attributed this lack of reliable fluorescent signal to two main reasons: (i) deficient (and potentially stochastic) expression of the gene(s) encoding the pH indicator(s) and (ii) some of the currently available ratiometric fluorescent protein indicators have been tailored for eukaryotic systems (Tournu *et al*., 2017; Reifenrath and Boles, 2018). In order to overcome these issues, we constructed a broad‐host‐range vector for the calibrated expression of a variant of the pH‐sensitive *pHluorin2* indicator protein (Mahon, 2011). pHluorin2 is a derivative of GFP that carries 11 point substitutions in its amino acid sequence, including the F64L mutation, known to enhance protein folding (Heim *et al*., 1995; Mahon, 2011). The sequence encoding pHluorin2 was codon‐optimized for *Pseudomonas* species, and a synthetic ribosome binding site was added to ensure sufficient expression, as indicated in *Experimental Procedures* (see also Sequence S1 in the Supporting Information). The synthetic module, which we termed *PHP* (**pH** indicator for ***P**seudomonas*), was placed under the transcriptional control of the constitutive EM7 promoter (*P*_EM7_) in the pSEVA2513 vector [kanamycin (Km)‐resistant, *oriV*(RSF1010)]. The resulting plasmid, termed pS2513·*PHP*, ensures the constitutive production of the PHP fluorescent protein in the bacterial cytosol regardless of the growth stage of the cells.

**Figure 1:**
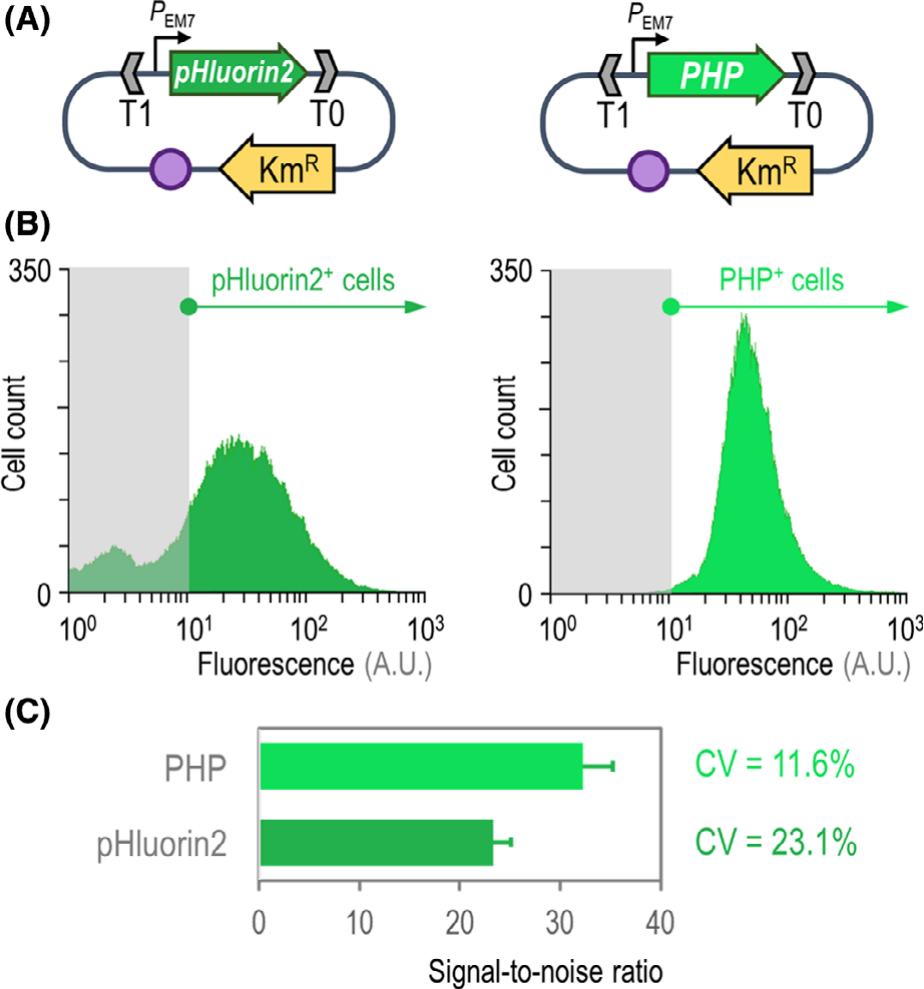
Benchmarking of the PHP indicator for the assessment of intracellular pH. A. Two separate expression plasmids were constructed for the calibrated expression of the synthetic genes encoding the pHluorin2 or PHP indicators. In both cases, the expression of the genes is driven by the constitutive *P*_EM__7_ promoter, and the backbone used for these constructs (vector pSEVA2513) carries the *oriV*(RSF1010) origin of replication, indicated in the scheme as a purple circle. The kanamycin‐resistance (Km^R^) determinant and the T1 and T0 transcriptional terminators are likewise highlighted in the plasmid maps. B. Flow cytometry exploration of the pHluorin2 and PHP indicators. Plasmid pS2513·*pHluorin2* or pS2513·*PHP* was transformed in *P*. *putida* KT2440, and the cells were grown in LB medium until the mid‐exponential phase of growth. Cells were processed as indicated in *Experimental Procedures*, and the histograms show the distribution of fluorescence (in arbitrary units, A.U.) stemming from pHluorin2 or PHP (the bacterial populations positive for the indicators are indicated in each case). The light grey rectangle in each plot identifies the region considered negative for the fluorescence signal, which was assessed with *P*. *putida* carrying the empty pSEVA2513 vector and grown under the same conditions. C. Key performance parameters of the pH indicators. The signal‐to‐noise ratio was calculated from flow cytometry measurements of *P*. *putida* KT2440 carrying either plasmid pS2513·*pHluorin2* or pS2513·*PHP* as indicated above. The bars represent the signal‐to‐noise ratio for each sensor and the 95% confidence interval calculated from four independent experiments. The coefficient of variation (CV) of the fluorescence signal was also calculated from these measurements.

We first set to compare the performance of the original pHluorin2 protein and the PHP indicator in *P*. *putida* KT2440 by comparing the fluorescence of the two proteins in individual bacterial cells by flow cytometry (Fig. 1). To this end, two identical, pSEVA2513‐based plasmid systems were used, in which the expression of either *pHluorin2* or *PHP* is driven by the same constitutive promoter under an equivalent vector copy number (Fig. 1A). These plasmids were individually transformed into *P*. *putida* KT2440, and the fluorescence stemming from either pHluorin2 or PHP was measured in exponentially growing cultures in LB medium by means of flow cytometry (Fig. 1B). Both the PHP and pHluorin2 fluorescence could be easily detected with the instrument settings used for GFP and related fluorescent proteins. As indicated by the histogram plots of cell count versus level of fluorescence of the indicators, cells expressing *pHluorin2* exhibited a bimodal distribution of fluorescence, with approximately 21% of the cell population displaying fluorescence levels similar to that of a negative control (i.e. *P*. *putida* KT2440 transformed with an empty pSEVA2513 vector). Cultures of strain KT2440 transformed with plasmid pS2513·*PHP*, in contrast, had a clearly unimodal fluorescence distribution, with > 99% of the bacterial population testing positive for the PHP signal. Moreover, the geometric mean value of fluorescence was considerably higher for PHP (fluorescence *x* mean = 42.5) than for the original pHluorin2 indicator (fluorescence *x* mean = 22.3). Next, we investigated the signal‐to‐noise ratio, a parameter reflecting the efficiency of fluorescence detection (Giesecke *et al*., 2017), for the two indicator proteins. The signal‐to‐noise ratio was 40% higher for the PHP indicator than for pHluorin2 (Fig. 1C) – an advantageous property that addresses one of the main problems previously experienced with fluorescent protein‐based pH indicators in *Pseudomonas* species. The coefficients of variation, a measure of the height versus width of fluorescence histograms and therefore an indication of the dispersion of the signal (Sharrow, 2001), were calculated for the two pH indicators according to the distribution of positive (i.e. pHluorin2^+^ or PHP^+^) cells of Fig. 1A. Again, the PHP protein outperformed pHluorin2, with a coefficient of variation which was approximately half of the original pHluorin2 indicator (Fig. 1C). Taken together, these results accredit the value of PHP as a uniformly accumulated fluorescent protein in *Pseudomonas*, with fluorescence characteristics similar to both GFP and pHluorin2.

**Figure 2:**
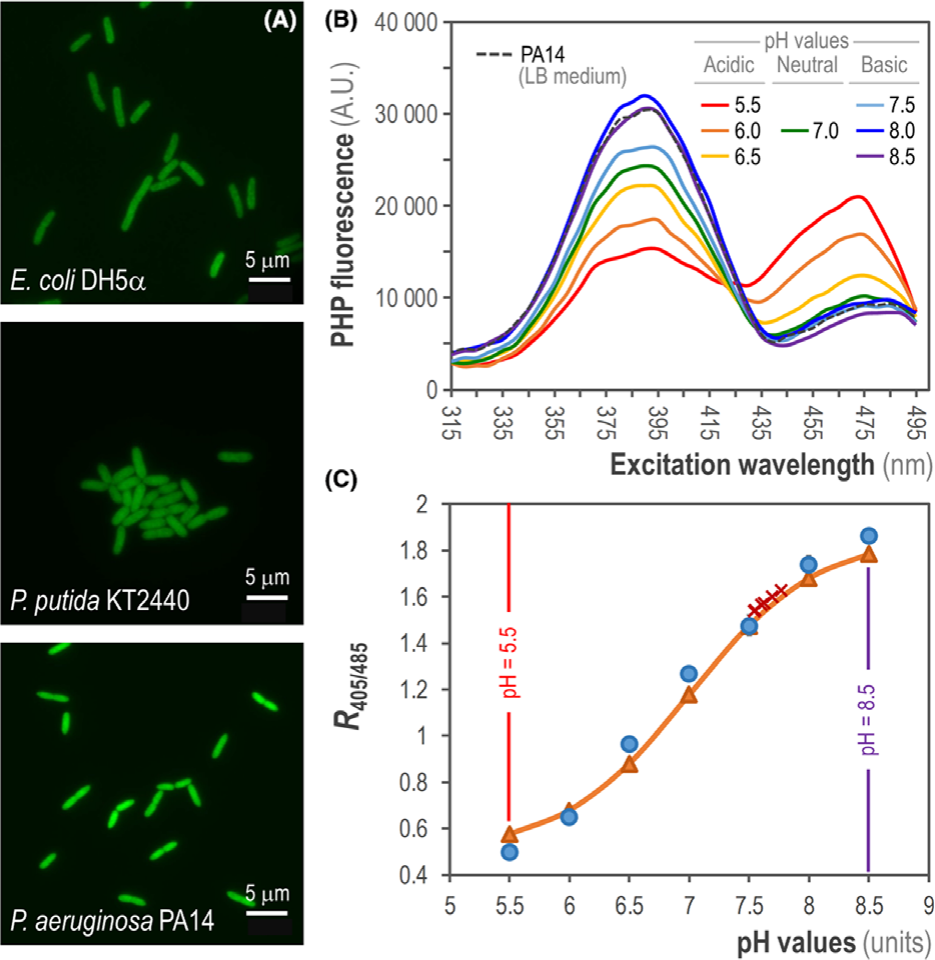
Assessment of intracellular pH (pH_i_) in different Gram‐negative bacteria. A. Fluorescent microscopy from exponentially growing cultures of *E*. *coli* DH5α, *P. putida* KT2440 and *P. aeruginosa* PA14 transformed with plasmid pS2513·*PHP* and grown in LB medium. Scale bars are indicated in each case. B. Fluorescence excitation spectrum of the PHP indicator in *P. aeruginosa* PA14/pS2513·*PHP* cultured in LB medium. The intracellular pH (pH_i_) of the cells was collapsed to the extracellular pH as outlined in *Experimental Procedures*. The mean values obtained from three independent experiments are shown. A.U., arbitrary units. C. Calibration curve of the PHP indicator in *P. aeruginosa* PA14/pS2513·*PHP* cells cultured in LB medium supplemented with 20 μM carbonyl cyanide *m*‐chlorophenyl hydrazine (CCCP). The data for the calibration curve were obtained as the ratio between the excitation peaks at λ_excitation_ = 405 nm and 485 nm (*R*_405/485_) plotted against the pH values of equilibrated cells (blue circles). These values were fitted using the Boltzmann sigmoid best‐fitting curve to obtain the calibration curve (orange triangles), which was used to calculate the pH_i_ values in *P*. *aeruginosa* PA14 during exponential growth (red crosses). The lowest and highest pH values tested (pH = 5.5 and 8.5) are highlighted. Data points are representative of independent triplicates, and the red crosses (corresponding to six individual determinations) are shown in the curve to indicate the narrow dispersion of experimentally calculated pH_i_ values.

Next, we experimentally verified the *PHP* expression by means of fluorescence microscopy of exponentially growing *E*. *coli* DH5α, *P. putida* KT2440 and *P*. *aeruginosa* PA14 carrying plasmid pS2513·*PHP* in LB medium. As observed in Fig. 2A, a homogeneous distribution of fluorescence was spotted across the individual cells of the three bacterial species examined, both during exponential growth and during the stationary phase – consistent with the results of flow cytometry experiments (Fig. 1). Subsequent tests in LB medium and M9 minimal medium indicated that the presence of plasmid pS2513·*PHP* did not affect the growth of *E*. *coli* or the two *Pseudomonas* species tested, neither in terms of specific growth rates nor the final cell density achieved in these cultures (data not shown). Moreover, flow cytometry analysis of the corresponding bacterial populations showed that the fluorescence signal followed a unimodal distribution in all three species both in complex and minimal culture media supplemented with glucose as the sole carbon source – indicating that the PHP protein can be used for studying pH_i_ across different culture conditions. The next question is how does the fluorescence stemming from PHP correlates with changes in pH_i_, as indicated below.

All ratiometric derivatives of GFP (e.g. pHluorin and pHluorin2) rely on the bimodality shown in their excitation spectra (Llopis *et al*., 1998; Esposito *et al*., 2008), which exhibit two distinct maxima at an excitation wavelength (λ_excitation_) ~ 395 nm and λ_excitation_ ~ 475 nm (Miesenböck *et al*., 1998; Mahon, 2011). We first aimed at scanning the excitation spectrum of the engineered PHP protein in *P*. *aeruginosa* PA14 cells harbouring plasmid pS2513·*PHP*. To this end, we recorded the culture's fluorescence emission at an emission wavelength (λ_emission_) = 515 nm in a standard microtiter plate fluorimeter. In order to check the effect of pH on the two excitation peaks of the PHP indicator, cells (previously grown in LB medium) were pelleted and resuspended in M9 minimal medium buffered at different physiologically relevant pH values (ranging from pH = 5.5 to pH = 8.5; see *Experimental Procedures* for details) in which the pH_i_ was collapsed to equalize the extracellular pH. This effect was achieved by the addition of benzoate (a permeant acid) and methylamine (a permeant base), and this pH collapse was experimentally verified by using the pH‐responsive, ratiometric chemical fluorophore 5(6)‐carboxyfluorescein diacetate succinimidyl ester (data not shown). We observed that upon acidification, the excitation peak of PHP at λ_excitation_ ~ 395 nm decreased with the corresponding increase in the peak at λ_excitation_ ~ 475 nm (Fig. 2B). Conversely, the excitation peak at λ_excitation_ ~ 395 nm increased when pH_i_ alkalized, while the intensity of the peak at λ_excitation_ ~ 475 nm concomitantly decreased. Almost identical spectra were obtained in *PHP*‐expressing *E*. *coli* DH5α and *P*. *putida* KT2440 (data not shown), confirming the expected PHP's sensitivity to changes in pH in the range of physiological pH values in different bacterial species. Although the bimodal pattern in both excitation peaks of PHP was essentially the same across the different Gram‐negative bacteria tested herein, we observed that the λ_excitation_ values of 405 nm and 485 nm reported by Martínez *et al*. (2012) gave rise to the same results regarding calculated pH_i_ values. We thus adopted these experimentally determined λ_excitation_ values (405 and 485 nm) for all subsequent experiments. In summary, our results thus far indicate that the spectral properties of the PHP fluorescent protein are very similar to those of pHluorin2, the parental ratiometric protein from which it derives (Mahon, 2011), across different bacterial species.

The next step was to use this dual excitation attribute of PHP to obtain a calibration curve by plotting the ratio between the fluorescence peaks at λ_excitation_ = 405 nm and λ_excitation_ = 485 nm (*R*_405/485_) against the pH values of equilibrated cells in the set of pH‐modified buffers described above. At first, we harvested exponentially growing *P. aeruginosa* PA14/pS2513·*PHP* cells incubated in LB medium. Once again, the pH_i_ of the cells was rapidly collapsed to equalize the pH of the different buffered solutions as detailed above. This allowed us to calculate highly reproducible sigmoid‐shape calibration curves using the Boltzmann best‐fitting equation as indicated in *Experimental Procedures* (Fig. 2C). The same procedure was applied to obtain calibration curves in cells sampled from exponentially growing cultures of *E*. *coli* DH5α/pS2513·*PHP* and *P. putida* KT2440/pS2513·*PHP* (Fig. S1A and S1B in the Supporting Information respectively), and of *P. aeruginosa* CH2682/pS2513·*PHP* (data not shown) in LB medium.

We observed some inconsistencies in the *R*_405/485_ values when the pH_i_ of *Pseudomonas* cells was collapsed in buffers with pH > 7.5. The differences in pH sensitivity of GFP‐borne indicators under alkaline conditions have been reported to be caused by the accessibility of chromophores to H^+^ ions in the solvent (Benčina, 2013). In order to overcome this problem, we set to supplement all *Pseudomonas* cultures with a sublethal amount of the hydrazone protonophore carbonyl cyanide *m*‐chlorophenyl hydrazine (CCCP). At low concentrations, this compound is known to translocate H^+^ ions across the cell wall, thereby dissipating the H^+^ gradient (i.e. ΔpH) across the cytoplasmic membrane without significantly compromising viability (Ghoul *et al*., 1989; Clark *et al*., 2015). Note that CCCP addition was necessary for *Pseudomonas* cultures as these species are known to display a very limited membrane permeability (see, for instance, the section below on antibiotic treatment). Protonophores (e.g. CCCP) increase membrane permeability by altering the electrochemical gradient, thus establishing an equilibrium across the cell membrane (Yu *et al*., 2015).

The addition of CCCP at 20 μM to *Pseudomonas* cultures tackled the problem observed during incubation of the cells in alkaline‐buffered solutions, and the presence of this protonophore did not impact cell growth across all the conditions tested herein, both in complex and minimal culture media. By applying this strategy, we could obtain highly reproducible sigmoid‐shape calibration curves for all *Pseudomonas* species harbouring plasmid pS2513·*PHP*, as shown for *P*. *aeruginosa* PA14 (Fig. 2C) and *P*. *putida* KT2440 (Fig. S1B in the Supporting Information). Moreover, addition of CCCP to *E*. *coli* cultures did not affect the calibration curves obtained for this species (data not shown), indicating that this strategy can be used in other Gram‐negative bacteria to collapse the pH_i_. With these tools and procedures at hand, we set to calculate pH_i_ in *E*. *coli* and in all *Pseudomonas* species under different culture conditions as indicated below.

### Physiological cytoplasmic pH values are conserved across Gram‐negative bacterial species

The calibration curves constructed for wild‐type *P. aeruginosa* strains (both PA14 and CH2682), *P. putida* KT2440 and *E*. *coli* DH5α were used to calculate the pH_i_ in actively growing cells cultured in LB medium (Table 1). Cell pellets of the four species, promptly harvested from the corresponding cultures, were resuspended in M9 medium salts lacking any permeant acid or base. Under these conditions, cells were able to maintain their pH_i_ homoeostasis. We observed that the pH_i_ values calculated for *E*. *coli* DH5α, *P. aeruginosa* PA14 and *P. putida* KT2440 were highly similar across all strains tested – with an average value of pH_i_ = 7.65 ± 0.14, close to neutrality. Furthermore, the pH_i_ values obtained correspond to those previously reported for neutrophilic bacteria using other detection methods, including fluorescent pH indicator proteins (Olsen *et al*., 2002; Sezonov *et al*., 2007; Krulwich *et al*., 2011; Martínez *et al*., 2012; Gao *et al*., 2016). A slightly higher pH_i_ value was observed in the CH2682 clinical isolate of *P. aeruginosa* (the cytoplasmic pH of this strain was consistently > 0.2 units above the values observed in the prototypical laboratory strain PA14). Conversely, the lowest pH_i_ value was observed for *E*. *coli* DH5α cells. The external pH values determined in the culture supernatants indicated that cells were able to maintain stable pH_i_ values while the external pH suffered changes (Table 1), e.g. due to the secretion of metabolic by‐products by the cells. A case in point is that of *E*. *coli*, for which the pH_i_ was approximately 1.5 units above the external pH. *E*. *coli* is known to secrete acetate and other low‐molecular‐weight organic acids to the culture medium (Nikel *et al*., 2008; Bernal *et al*., 2016), which is probably the reason explaining the acidification of the extracellular medium in these cultures. All the cultures of *Pseudomonas* species, in contrast, had pH values close to neutrality – displaying differences between pH_i_ and extracellular pH smaller than in *E*. *coli*.

**Table 1:** Intracellular pH measurements of selected Gram‐negative bacteria growing in LB medium

Bacterial strain	Relevant characteristics	Specific growth rate (h^−1^)	Cytoplasmic pH	Extracellular pH
*E*. *coli* DH5α	Laboratory strain typically used for cloning	1.46 ± 0.05	7.54 ± 0.11	6.05 ± 0.02
*P. putida* KT2440	Wild‐type strain, platform bacterial host	1.89 ± 0.06	7.77 ± 0.11	7.51 ± 0.01
*P. aeruginosa* PA14	Wild‐type strain, wide host virulence spectrum	0.92 ± 0.02	7.63 ± 0.08	7.12 ± 0.02
*P. aeruginosa* CH2682	Wild‐type strain, clinical isolate	0.57 ± 0.03	7.87 ± 0.07	7.54 ± 0.02

All strains, carrying plasmid pS2513·*PHP*, were grown in LB medium, and the cytoplasmic pH was determined during exponential growth. The pH of the LB medium was determined to be 7.05 ± 0.02 just prior to inoculation of the cultures. These results represent the mean value of each parameter ± standard deviation of triplicate measurements from at least three independent experiments.

In order to validate these results against a different protocol for determination of pH_i_, we calculated the pH values of both *P*. *aeruginosa* PA14 and *P*. *putida* KT2440 by applying the well‐established method of staining the cells with the pH‐responsive fluorophore 5(6)‐carboxyfluorescein diacetate succinimidyl ester (Riondet *et al*., 1997). Exponentially growing cells of each species cultured in LB medium were harvested and treated with the chemical fluorophore as indicated in *Experimental Procedures*. The pH_i_ values calculated with this method (pH_i_ *P*. *aeruginosa* PA14 = 7.78 ± 0.19 and pH_i_ *P*. *putida* KT2440 = 7.59 ± 0.12) had a variation of < 3% with respect to those obtained *via* the PHP indicator – yet, the dispersion of experimental pH determinations across replicates of the same experiment was, on average, higher for the cells treated with 5(6)‐carboxyfluorescein diacetate succinimidyl ester than for the cells carrying pS2513·*PHP*. Taken together, these results demonstrate that PHP is a reliable indicator of pH_i_ in *Pseudomonas* species, indicating that cells tightly maintain a pH_i_ when cultured in favourable conditions (i.e. fast growth in a rich culture medium), and the next question was to evaluate how they respond to internal and external metabolic perturbations in terms of pH homoeostasis.

### The cytoplasmic pH of *P. putida* fluctuates depending on the glucose consumption routes

In the model platform bacterium *P. putida* KT2440 (Benedetti *et al*., 2016; Nikel and de Lorenzo, 2018; Calero and Nikel, 2019), the initial steps for the catabolism of glucose occur simultaneously through the phosphorylation of the sugar to glucose‐6‐*P* by the enzyme glucokinase (Glk), or by its oxidation to gluconate by means of glucose 2‐dehydrogenase (Gcd) (del Castillo *et al*., 2007; Nikel and de Lorenzo, 2014; Nikel *et al*., 2015; Nikel and Chavarría, 2016). The oxidation of glucose in the periplasm yields low‐molecular‐weight organic acids, e.g. gluconate and 2‐ketogluconate (Hirshfield *et al*., 2003; Sánchez‐Pascuala *et al*., 2019). Hence, we set to study how the use of this peripheral pathway influences the pH_i_ in strain KT2440 and its glycolytic mutant derivatives. We transformed the pS2513·*PHP* plasmid into two *P*. *putida* mutants, each devoid of one of the two aforementioned enzymes, and calculated the pH_i_ in exponential and stationary phase cultures grown in M9 minimal medium containing glucose as the only carbon source (i.e. glycolytic conditions). In a Δ*glk* mutant of strain KT2440, the bulk of hexoses (80–90%) is oxidized into gluconate, whereas in a Δ*gcd* mutant, sugars are exclusively phosphorylated to glucose‐6‐*P* (Dvořák *et al*., 2017; Sánchez‐Pascuala *et al*., 2017, 2019). Once again, cultures of *P. putida* KT2440 grown in the aforementioned medium supplemented with CCCP were used to calculate the calibration curve for pH_i_ quantification in all strains (Fig. S2 in the Supporting Information).

**Figure 3:**
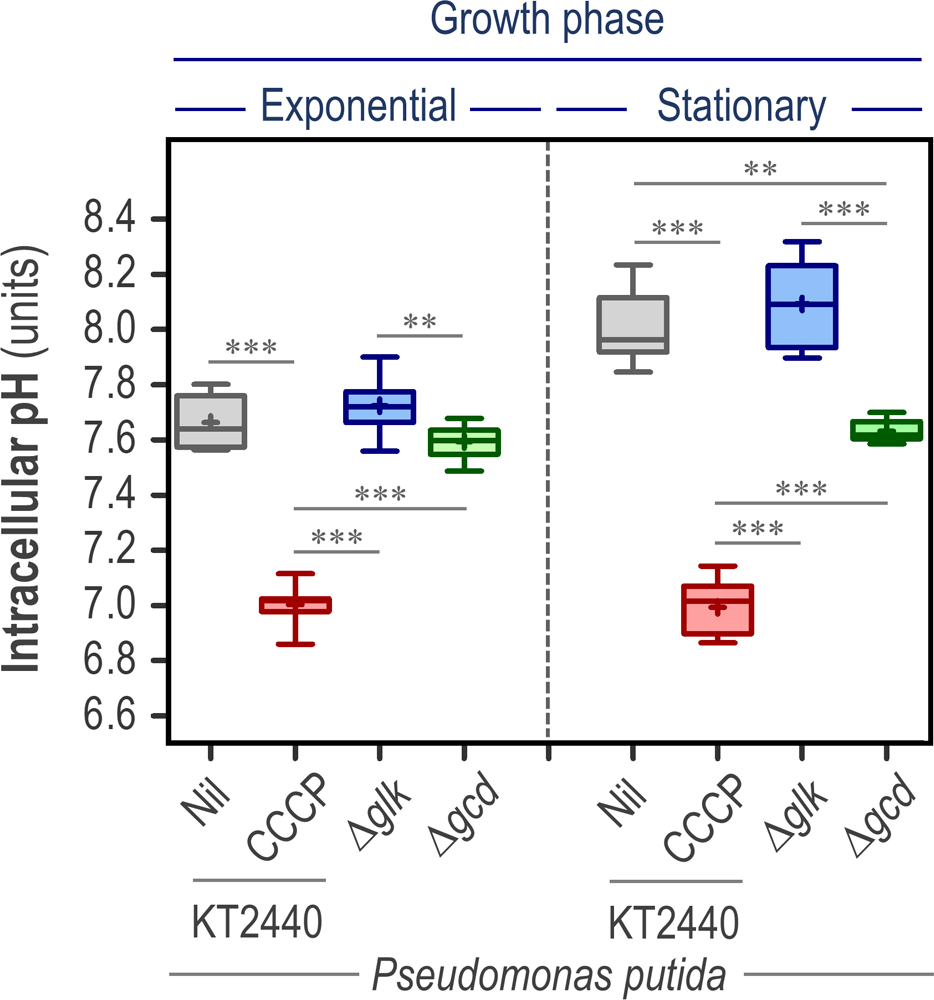
The cytoplasmic pH of *P*. *putida* is altered by the pathway used for glucose consumption. Wild‐type *P. putida* KT2440 and its isogenic mutants in the enzymes glucokinase (Δ*glk*) and glucose 2‐dehydrogenase (Δ*glk*) were transformed with plasmid pS2513·*PHP* and grown in M9 minimal medium supplemented with glucose at 20 mM as the sole carbon source. The intracellular pH (pH_i_) was investigated during the exponential and stationary (24 h post‐inoculation) phase of growth. The pH_i_ of wild‐type cells cultured in the presence of 20 μM carbonyl cyanide *m*‐chlorophenyl hydrazine (CCCP) is also shown in the figure. Independent triplicates of the experiments were carried out, and the level of statistical significance in the pairwise comparisons indicated in the comparisons across experimental conditions is indicated as ** *P *<* *0.01 and *** *P *<* *0.001 (ANOVA).

As indicated in Fig. 3, cytoplasmic pH values calculated for exponentially growing *P. putida* KT2440 in M9 minimal medium with glucose (pH = 7.66 ± 0.09) were similar to the values calculated when the same bacteria were grown in LB medium (Table 1). Additionally, the cytosolic pH of wild‐type cells grown in the presence of CCCP showed a drop in pH_i_, decreasing to levels approaching the extracellular culture medium (i.e. around neutrality). This pH_i_ range was observed to be maintained around the same values along the growth curve, even after the cultures reached the stationary phase (after 24 h of cultivation; see Fig. 3). The PHP indicator thus captured the increase in pH_i_ as cells transitioned from exponential growth into the stationary phase, a well‐described phenomenon in Gram‐negative bacteria (Lambert *et al*., 1997; Kurbatov *et al*., 2006; Reva *et al*., 2006).

Interestingly, we observed significant differences in the cytoplasmic pH between the Δ*glk* and Δ*gcd* derivatives of *P. putida* (Fig. 3). The Δ*glk* strain had a slightly higher (albeit statistically significant) pH_i_ than the Δ*gcd* mutant during exponential growth; this difference became more evident during stationary phase (e.g. pH_i_ = 8.11 for the Δ*glk* strain and pH_i_ = 7.61 for the Δ*gcd* strain after 24 h of cultivation). Forcing the carbon flow through oxidation (in the Glk‐deficient strain) leads to the generation of gluconate (and 2‐ketogluconate, the further oxidized product of this metabolic branch of hexose processing). Gram‐negative bacteria, including *Pseudomonas* species, are known to respond to slight acidic stress by increasing their pH_i_ (Krulwich *et al*., 2011), and this general response could partially account for the results. In addition, the ΔpH (and PMF), which is another determining factor of the differences in pH_i_ across the strains tested (Casey *et al*., 2010), is likewise expected to be affected in these mutants. In turn, the differences in ΔpH result in an alteration of the energetic status of the cells (Fuhrer *et al*., 2005; Voskuil *et al*., 2018). Interestingly, the Gcd‐deficient *P*. *putida* strain was able to stably maintain pH_i_ values around neutrality during the whole cultivation period. Experimental determination of gluconate and 2‐ketogluconate confirmed that there were no detectable acidic by‐products secreted into culture supernatants, while cultures of both the wild‐type strain and its Δ*glk* derivative reached gluconate and 2‐ketogluconate concentrations of approximately 5 and 1.5 mM and 7.8 and 2.9 mM respectively. This phenomenon could account for the stable pH_i_ values observed in the Δ*gcd* mutant, which would not be subjected to acidic stress caused by the secretion of metabolic by‐products from glucose oxidation. The extracellular pH was assessed in all these cultures (Table S1 in the Supporting Information), providing further support to this notion: cultures of both the wild‐type strain and the Δ*glk* derivative had an acidic external milieu (in particular, for the later strain), whereas the extracellular pH in cultures of the Gcd‐deficient variant was maintained around neutrality values. When cultures of all the three strains were grown in M9 minimal medium containing succinate as the sole carbon source (i.e. under gluconeogenic conditions), no major differences were observed in pH_i_, neither during exponential growth nor during the stationary phase (in both cases, the average pH_i_ = 7.35 ± 0.18 for all the strains tested) – thus indicating that the differences observed in pH_i_ values in the glucose cultures could be attributed to the use of different metabolic routes deployed for sugar utilization. While *endogenous* (i.e. metabolic) perturbations have been shown to exert a strong effect on pH homoeostasis (and hence, in the pH_i_ values) in these *Pseudomonas* strains, we next explored how these parameters are affected by *external* perturbations, such as a sudden pH shift and treatment with antimicrobial agents, as indicated in the sections below.

### The PHP indicator can be used to track time‐dependent changes in cytoplasmic pH upon a shift in extracellular pH

**Figure 4:**
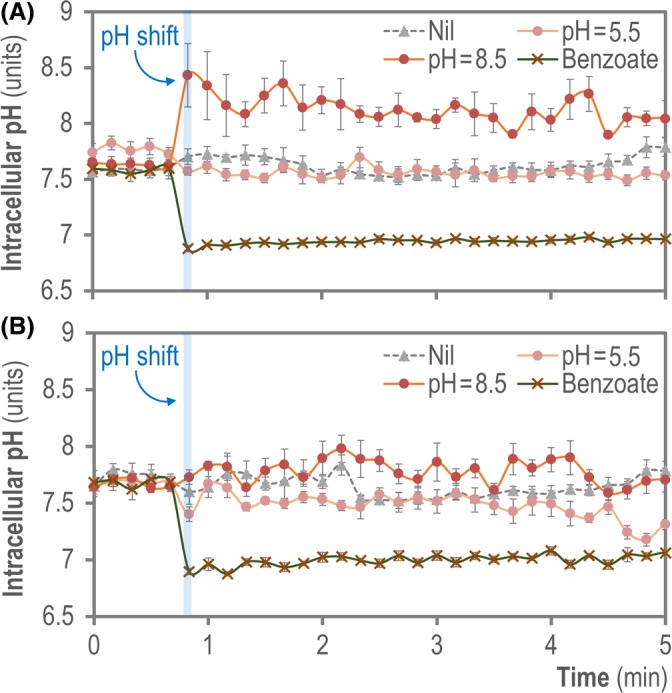
Monitoring the dynamic changes of cytoplasmic pH upon external pH shifts. Exponentially growing cells of (A) *E*. *coli* MG1655/pS2513·*PHP* or (B) *P. putida* KT2440/pS2513·*PHP* cultured in M9 minimal medium containing 20 mM glucose were aliquoted into a 96‐well microtiter plate and the cytoplasmic pH was continuously monitored, before and after a shift in the extracellular pH was induced by the addition of the same culture medium buffered at pH = 5.5 or pH = 8.5 as indicated in the *Experimental Procedures*, or sodium benzoate (permeant acid). Changes in cytoplasmic pH were monitored every 10 s during the first 5 min of incubation. Data shown in each case indicate mean values from independent triplicates ± SD.

We investigated if the PHP indicator could reflect adaptive changes in pH_i_ upon a sudden shift in extracellular pH values, both acidic and alkaline. To this end, both wild‐type *E*. *coli* MG1655 and *P*. *putida* KT2440 harbouring plasmid pS2513·*PHP* were grown in M9 minimal medium containing glucose, and the cell suspensions were challenged by exposure to a buffer at pH = 5.5 or pH = 8.5 (see *Experimental Procedures* for details). The pH_i_ values were calculated from fluorescence readings taken every 10 sec over 5 min to capture the short‐term dynamics of cytoplasmic pH homoeostasis upon the acidic or alkaline challenge (Fig. 4). Control experiments were run for both bacterial species by adding benzoic acid to the cell suspension in order to collapse pH_i_ – an operation which, as expected, resulted in an almost immediate drop in pH_i_ to match the pH value of the external medium (i.e. pH = 6.95 ± 0.05). The pH_i_ values recorded for *E*. *coli* and *P*. *putida* did not recover after exposure to sodium benzoate (nor did they recover during 1 h post‐treatment, data not shown). In experiments without any perturbation in extracellular pH, pH_i_ values were stably maintained during the assay within the values previously observed for these bacteria (i.e. in the range of 7.5–7.8 units; see also Table 1). When *E*. *coli* was challenged with a buffer at pH = 5.5, the cells did not react with a sudden drop in pH_i_, in contrast with the response to a pH = 8.5 shift, which entailed a sudden increased in pH_i_, followed by a slow, steady decrease of the pH_i_ to the values pre‐shift (Fig. 4A). From 9 min onwards, the pH_i_ of base‐challenged cells matched that of untreated control cultures (data not shown). Overall, these observations are similar to the results previously reported by Padan *et al*. (2005) and Šeputienė *et al*. (2006), indicating that the cytoplasmic pH of *E*. *coli* K‐12 is more sensitive to alkaline challenges than to acidification of the surrounding culture medium. The pattern of pH_i_ values in *P*. *putida* KT2440 upon pH shifts was much more rigid in comparison (Fig. 4B). Exposure of the bacterial cells to pH = 5.5 or pH = 8.5 did not result in any major change in pH_i_ over the whole experimental period, and the fluctuations observed in PHP fluorescence (hence, in pH_i_) were within the range of the experimental error for these determinations. Taken together, the results of these experiments reflect the increased membrane permeability of *E*. *coli* as compared to that in *P*. *putida*. In fact, the outer membrane of several *Pseudomonas* species is known to exhibit from 100‐ to 400‐fold lower permeability to hydrophilic compounds than that of *E*. *coli* (Yoshimura and Nikaido, 1982; Llamas *et al*., 2000; Sohlenkamp, 2017). Our results are also in line with the observations reported by Reva *et al*. (2006), suggesting that *P*. *putida* KT2440 displays increased resistance to pH shifts as compared to Gram‐negative Enterobacteria. Since the PHP indicator enabled to follow changes in pH_i_ upon alkalinization or acidification of the external milieu, we also explored if this procedure could be used to capture the dynamic changes in pH_i_ brought about by antibiotic treatment as disclosed below.

### Response of the cytoplasmic pH of *P. aeruginosa* to treatment with different antibiotics

**Figure 5:**
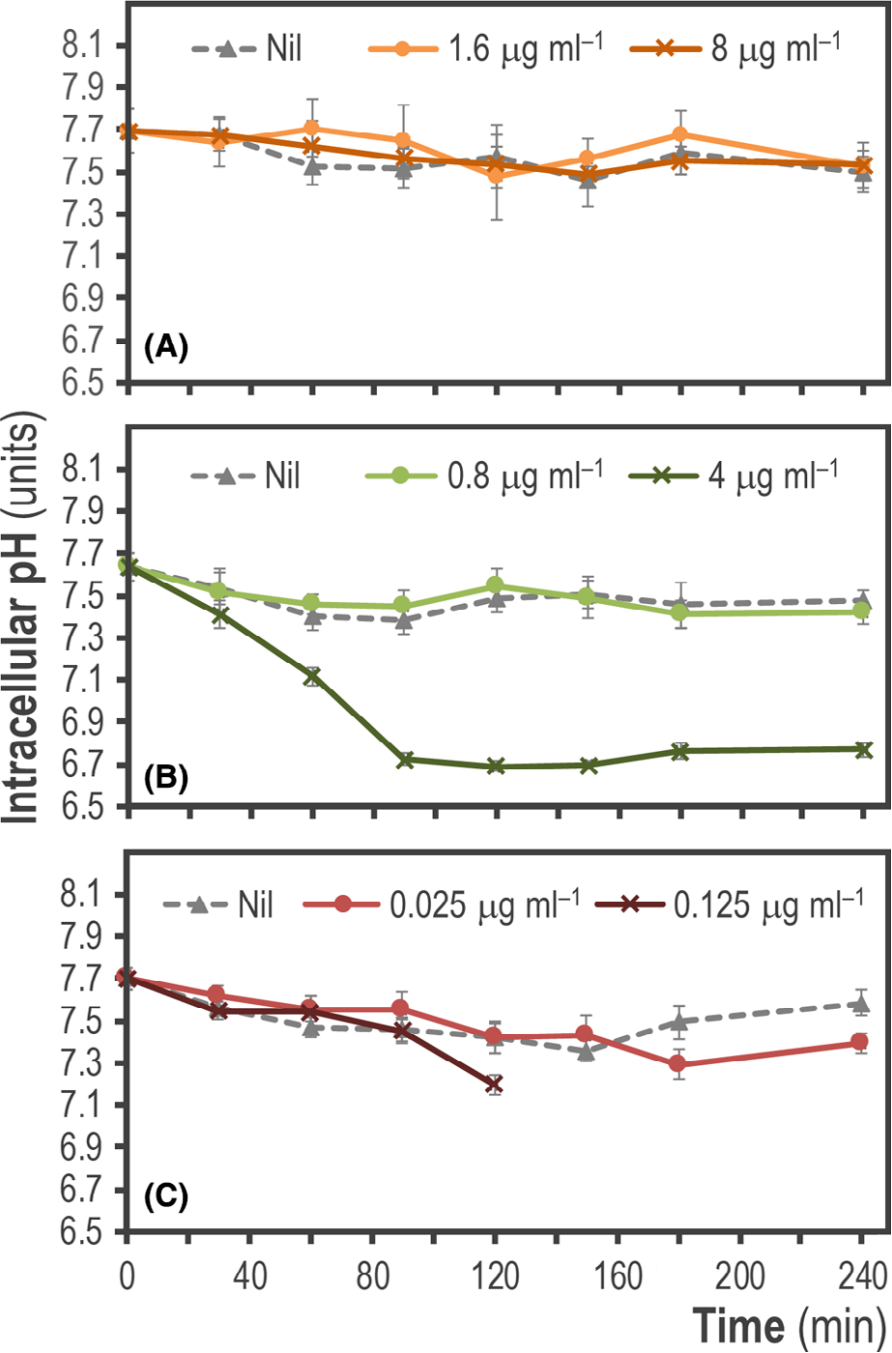
Monitoring the changes of intracellular pH (pH_i_) in *P. aeruginosa* PA14 upon treatment with different antibiotics. Exponentially growing cultures of *P. aeruginosa* PA14 transformed with plasmid pS2513·*PHP* and grown in LB medium were challenged with one‐fifth (0.2×) and full (1×) minimal inhibitory concentrations of (A) ceftazidime (CAZ), (B) amikacin (AMK) and (C) ciprofloxacin (CIP) as indicated in the graphs. The data shown in each case indicate mean values of pH_i_ from independent triplicates ± SD.

The next phenotype that we addressed was the changes of pH_i_ due to addition of clinically relevant antibiotics often used for the treatment of *P*. *aeruginosa* infections. To this end, we first grew *P. aeruginosa* PA14 harbouring plasmid pS2513·*PHP* in LB medium to reach the mid‐exponential phase of growth [i.e. optical density at 600 nm (OD_600_) = 0.4–0.5]. At this point, we treated the cultures with three different antibiotics at one‐fifth (0.2×) or at full (1×) minimal inhibitory concentrations (MIC) of the antibiotics, and we followed the changes in pH_i_ continuously over the course of 4 h (Fig. 5). The antibiotics chosen for this purpose belong to three different functional classes based on their mode of action: ceftazidime (CAZ, a cephalosporin), amikacin (AMK, an aminoglycoside) and ciprofloxacin (CIP, a fluoroquinolone) (Lambert, 2002; Morita *et al*., 2014; Bassetti *et al*., 2018).

No significant changes in the cytosolic pH were observed after exposure of *P. aeruginosa* PA14 to 0.2 × (1.6 μg ml^−1^) and 1 × (8 μg ml^−1^) MIC concentrations of CAZ when compared to untreated cells (Fig. 5A). However, a clear decrease in the pH_i_ values was observed after the treatment of the same cells with AMK, albeit only when the antibiotic concentration matched the full MIC (4 μg ml^−1^, Fig. 5B). The drop in pH_i_ could be observed very early on (becoming noticeable just 1 h after treatment) and was retained until the end of the experiment. Remarkably, the pH_i_ in cells exposed to 1 × MIC concentration of AMK dropped to levels close to pH = 6.7. CIP also caused a reduction in the pH, which was evident from 2 h post‐antibiotic treatment with 1 × MIC (0.125 μg ml^−1^), and after 3 h when cells were challenged with 0.2 × MIC (0.025 μg ml^−1^, Fig. 5C). This decrease was, however, not as strong as the pH drop observed in AMK‐treated cultures. Interestingly, it was not possible to continue the pH_i_ assessment in cultures amended with 1 × MIC of CIP beyond 2 h after treatment. In this particular case, the integrity of the cells was likely compromised due to the harsh antimicrobial treatment, making it very difficult to obtain consistent cell pellets after centrifugation of the bacterial suspension. This phenomenon has been reported for *P*. *aeruginosa* when exposed to different antibiotics, including treatment with CIP (Day *et al*., 1993; Lee *et al*., 2017).

The results above shed light on the mechanism of action of antibiotics on *P*. *aeruginosa* cells, an area of active research. Bartek *et al*. (2016) suggested that antibiotic exposure with different types of bactericidal compounds increases the cytosolic pH in *Mycobacterium smegmatis*. Furthermore, they proposed that there is a general mechanism by which antibiotics kill bacteria through disruption of cellular biosynthetic processes (caused by the primary inhibition of either nucleic acids, proteins or cell wall synthesis) which in turn drives an imbalance in H^+^ homoeostasis leading to a potentially lethal increase in the pH_i_. In our experiments, we have used three different types of antibiotics, both at MIC and sub‐MIC concentrations. However, we could not observe an increase in pH_i_ along the 4 h of antibiotic treatment. Conversely, exposure to high concentrations of AMK or CIP caused a drop in the cytosolic pH (Fig. 5). This effect could be caused by the permeabilization of the cell membrane due to a physiological response to the damage caused by the aforementioned antibiotics, a phenomenon previously characterized in Gram‐negative bacteria (Dougherty and Saukkonen, 1985; Davis, 1987; Novo *et al*., 2000). The differential permeability of the cell membrane of *Pseudomonas* species to chemicals is a decisive factor that also dictates tolerance or sensitivity to antimicrobials (Novo *et al*., 2000; Shen *et al*., 2019). On the other hand, the phenomenon of pH increase upon antibiotic treatment could be affected by the nature of the bacteria tested [e.g. Gram‐positive versus Gram‐negative species (Eichenberger and Thaden, 2019)], and also the experimental conditions used (e.g. addition of antibiotics at the onset of the cultivation or during active growth of the bacterial cells). Taken together, our results suggest that *P*. *aeruginosa* may react differently to treatment with diverse types of antibiotics, differing in their mechanism of action, at the level of pH_i_ homoeostasis.

## Conclusions

**Figure 6:**
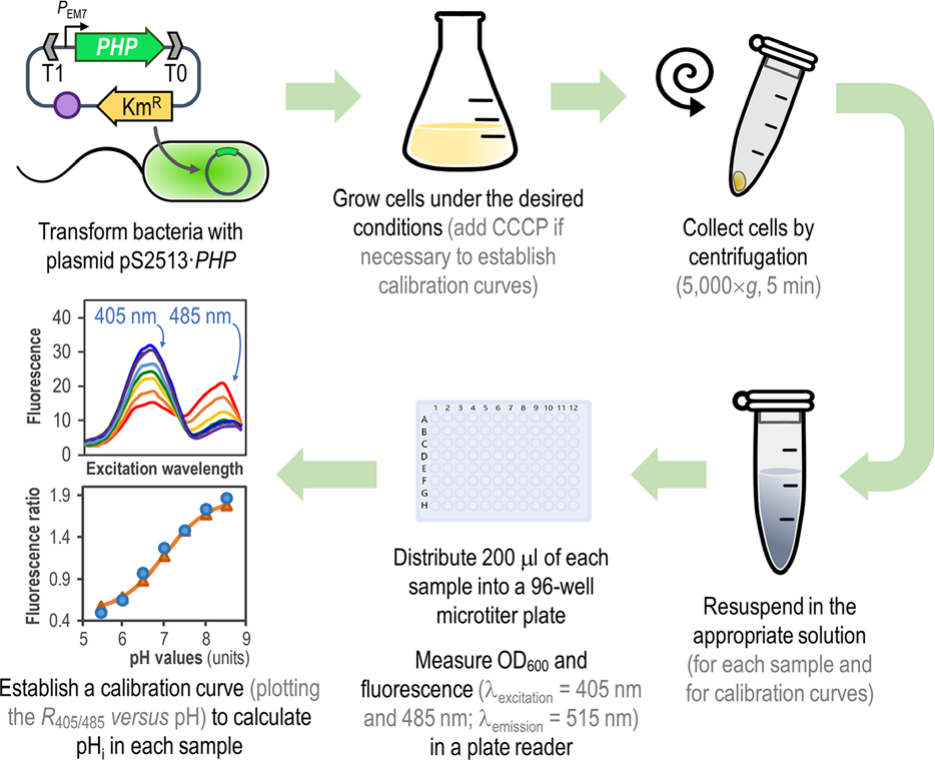
General procedure for *in vivo* assessment of intracellular pH (pH_i_) in *Pseudomonas* species by means of the PHP indicator. Plasmid pS2513·*PHP* is introduced into the bacteria of interest by chemical transformation, mating or electroporation. The expression of *PHP* is driven by the constitutive *P*_EM__7_ promoter, and vector pSEVA2513 carries the *oriV*(RSF1010) origin of replication, indicated as a purple circle. The kanamycin‐resistance (Km^R^) determinant and the T1 and T0 transcriptional terminators are likewise highlighted in the plasmid. Cells are grown in the appropriate culture medium, and a small culture sample is harvested by centrifugation. The cell pellet is resuspended in M9 medium salts to a final optical density at 600 nm (OD_600_) = 0.4–0.5. To establish the calibration curve, a second culture is prepared in the same culture medium supplemented with carbonyl cyanide *m*‐chlorophenyl hydrazine (CCCP). Cells from this culture are also harvested by centrifugation and equilibrated in M9 medium salts adjusted to different pH values (ranging from pH = 5.5–8.5, see *Experimental Procedures*) to a final OD_600_ = 0.4–0.5. A 200‐μl aliquot of each cell suspension is transferred into a 96‐well microtiter plate, and OD_600_ and fluorescence (λ_excitation_ = 405 nm and 485 nm; λ_emission_ = 515 nm) are measured in a plate reader. Finally, the *R*_405/485_ values are plotted versus the pH values of pH‐equilibrated cells (calibration curve), and the pH_i_ is calculated using a Boltzmann sigmoid best‐fitting equation.

In this work, we described the construction and calibration of a non‐invasive pH_i_ indicator and we have demonstrated its efficacy in *E*. *coli* and *Pseudomonas* species by using a standard protocol that can be easily reproduced in different bacteria (Fig. 6). We have shown the efficacy of this new tool in *P. aeruginosa* and *P. putida* by quantifying the alterations in pH_i_ homoeostasis elicited after internal (redirection of glucose consumption pathways) and external (antibiotic treatment) metabolic perturbations, as well as pH shifts for both *E*. *coli* and *P*. *putida*. Although the vector platform containing the PHP indicator was primarily designed for *Pseudomonas* species*,* it can be easily adapted to other Gram‐negative bacteria (unlike other pH ratiometric protein‐bearing plasmids), since our system was constructed following the rules set in the *SEVA* format (Silva‐Rocha *et al*., 2013). Plasmids constructed according to this format allow for the easy exchange of different parts at the user's will. Unique *oriV* modules can be swapped to further broaden the (already sizeable) host range of the plasmids. Typically, different intracellular amounts of PHP will be expected by increasing or decreasing the plasmid copy number. Other manipulations are also possible: different antibiotic markers can be exchanged into the vector depending on the resistance profile of the bacterium under scrutiny. Simple approaches for the online assessment of intracellular properties such as pH_i_ will thus enable both fundamental and applied studies, e.g. biosensor‐based evolution of phenotypic traits (Fernández‐Cabezón *et al*., 2019).

## Experimental procedures

### Bacterial strains and culture conditions

All strains used in this work are derivatives of *E*. *coli* strains MG1655 (Blattner *et al*., 1997) and DH5α (Manoil and Beckwith, 1985), *P. putida* KT2440 (Bagdasarian *et al*., 1981; Belda *et al*., 2016), and *P. aeruginosa* strains PA14 (Friedman and Kolter, 2004) and CH2682 (Hornischer *et al*., 2018). *E*. *coli* DH5α was used both for the propagation and construction of plasmids and for pH_i_ calculations, along with the well‐characterized K‐12 strain MG1655 (Blattner *et al*., 1997). The *P. putida* Δ*glk* and Δ*gcd* mutants are derivatives of the parental strain KT2440 with specific mutations in the genes encoding the enzymes that execute the first steps of glucose utilization (Sánchez‐Pascuala *et al*., 2017, 2019). *P. aeruginosa* CH2682 is a meropenem‐resistant clinical strain isolated from a patient with a rectal infection (Hornischer *et al*., 2018).

Unless indicated otherwise, bacteria were cultured in 50‐ml Erlenmeyer flasks containing 10 ml of LB medium (Green and Sambrook, 2012) with agitation at 180 rpm at either 30°C (*P*. *putida* strains) or 37°C (*E*. *coli* and *P*. *aeruginosa* strains). In some experiments, *P. putida* strains were also grown in 10 ml of M9 minimal medium, containing 8.5 g l^−1^ Na_2_HPO_4_·2H_2_O, 3 g l^−1^ KH_2_PO_4_, 1 g l^−1^ NH_4_Cl, 0.5 g l^−1^ NaCl, 0.5 g l^−1^ MgSO_4_·7H_2_O and 2.5 ml l^−1^ of trace element solution (Abril *et al*., 1989; Nikel and de Lorenzo, 2013) with glucose as the sole carbon source at 3.6 g l^−1^ (i.e. 20 mM). *P. putida* strains were also grown under gluconeogenic conditions by using succinate at 20 mM as the sole carbon source. Km was added when needed to ensure plasmid maintenance either at 50 μg ml^−1^ (for *E*. *coli* strains and *P. putida* KT2440 and its derivatives) or 500 μg ml^−1^ (for all *P. aeruginosa* strains). Additionally, the MIC of the antibiotics CAZ, AMK and CIP was determined in *P. aeruginosa* strains using macrodilutions in Mueller–Hinton broth as described previously (Wiegand *et al*., 2008).

For growth rate measurements, overnight pre‐cultures of all the strains were grown for 16 h in 3 ml of LB medium supplemented with Km. One millilitre of each pre‐culture was harvested by centrifugation (5000 *g*, 5 min) and washed twice with 1 ml of fresh LB. The cells were resuspended in fresh LB medium containing Km, diluted to a starting OD_600_ = 0.02 and distributed (200 μl each well) into a *Honeycomb 2* ^TM^ plate (Thermo Fisher Scientific, Waltham, MA, USA). The plate was incubated into an automated *Bioscreen C* MBR™ plate reader (Oy Growth Curves Ab, Helsinki, Finland), and the growth of each sample was constantly monitored by measuring its OD_600_. The growth rates of each strain were calculated from the exponential fraction of the growth curves (Nikel and Chavarría, 2016). The extracellular pH of culture media was measured in 1‐ml aliquots of culture broth harvested at the times indicated, after separation of the bacterial biomass by centrifugation (5000 *g*, 5 min) and filtering the supernatant with a 0.2‐μm filter‐membrane (Filtropur S0.2; Sarstedt, Nümbrecht, Germany), with a *SevenCompact*™ pH meter S210 system (Metler‐Toledo A/S, Glostrup, Denmark). In experiments involving *P*. *putida* and its glycolytic mutant derivatives, the concentration of gluconate and 2‐ketogluconate in culture supernatants was determined by high‐pressure liquid chromatography as explained by Nikel *et al*. (2015).

### Construction of a vector for the constitutive expression of the gene encoding the PHP indicator

The DNA sequence of the *pHluorin2* gene was codon‐optimized for *P*. *putida,* and the resulting coding sequence was ordered as a synthetic DNA fragment (Integrated DNA Technologies, Leuven, Belgium). For the sake of clarity, we refer to the optimized sequence as *PHP* throughout the text. The entire *PHP* sequence was PCR‐amplified from the synthetic DNA with primers 5′‐AAA AAG CTT *AGG AGG AAA AA*C AT**A TG**A GCA‐3′ and 5′‐TTT ACT AGT TTA CTT GTA GAG TTC ATC CAT ACC‐3′ containing the restriction sites HindIII and SpeI (underlined) and a ribosome binding site (italics) adjacent to the start *ATG* codon, indicated in boldface in the forward primer (see also Sequence S1 in the Supporting Information). The PCR product and the expression vector pSEVA2513 were double‐digested with the restriction enzymes HindIII and SpeI and column purified (*QIAquick*™ PCR purification kit; Qiagen, Hilden, Germany). Both DNA fragments were ligated to give rise to plasmid pS2513·*PHP*, used throughout this study for pH_i_ determinations. The correct plasmid sequence was confirmed by sequencing of plasmid DNA retrieved from selected *E*. *coli* DH5α clones. Plasmid pS2513·*PHP* was later introduced into *P. putida* and *P. aeruginosa* strains by electroporation (Choi *et al*., 2006; Volke *et al*., 2019; Wirth *et al*., 2019). Plasmid pS2513·*PHP* has been deposited in AddGene (https://www.addgene.org/) with catalogue # 122590.

### Fluorescence microscopy of bacterial cells expressing PHP

Overnight cultures of bacteria transformed with plasmid pS2513·*PHP* were grown in LB medium supplemented with Km at the appropriate concentrations (see above). Next, 1 ml of each pre‐culture was pelleted by centrifugation (5000 *g*, 5 min) and washed twice with 1 ml of fresh LB. Erlenmeyer flasks (50 ml) filled with 10 ml of fresh LB medium (without Km) were inoculated with the washed cells to an OD_600_ of 0.05 and grown until mid‐exponential phase (OD_600_ = 0.4–0.5). Cells were fixed by mixing an aliquot of the culture with 5% (v/v) paraformaldehyde in a 1:1 (v/v) ratio, followed by a 30 min incubation at room temperature. Finally, 10 μl of the cell suspensions were placed onto 1.5% (w/v) agarose pads as described by Young *et al*. (2011) and visualized with a 100 × immersion oil objective in a Nikon *Eclipse*™*Ti* microscope (Nikon Instruments, Amsterdam, Netherlands) equipped with a eGFP·ET filter set and a Hamamatsu *Orca* Flash 4.0 camera (Hamamatsu Photonics K.K., Shizuoka, Japan). Fluorescence images were analysed with the Fiji software package (Schindelin *et al*., 2012).

### *In vivo* assessment of cytoplasmic pH by flow cytometry and fluorescence spectroscopy using the pHluorin2 or PHP indicators and fluorescent chemical probes

Overnight grown pre‐cultures of each bacterium carrying plasmid pS2513·*PHP* or plasmid pS2513·*pHluorin2* were prepared in the indicated culture media (LB or M9 minimal medium, as specified in each case) added with the necessary antibiotics as described above for the fluorescence microscopy experiments. Unless stated otherwise, 10 ml cultures of each strain were prepared in the same media devoid of antibiotics and grown until mid‐exponential phase (OD_600_ = 0.4–0.5). Next, 1 ml aliquots were taken from these cultures, and the OD_600_ of each sample was adjusted (if necessary) to 0.4 before centrifugation (5000 *g*, 5 min) and cell resuspension in 1 ml of M9 salts devoid of any carbon source, which yielded a cell density of approximately 10^5^ cells ml^−1^. For flow cytometry experiments, the resulting cell suspension was directly subjected to analysis in a MACSQuant VYB flow cytometer (Miltenyi Biotec, Bergisch Gladbach, Germany). Both pHluorin2 and PHP were assessed by using the same instrument settings. The fluorescence emission at 525 nm was detected using a 525/40‐nm band pass filter array upon excitation with a diode‐pumped solid‐state laser at 488 nm (Nikel *et al*., 2016). Size‐related forward scatter signals gathered by the cytometer were used by the *FlowJo* software v. 9.6.2 (FlowJo LLC, Ashland, OR, USA) to calculate the geometric mean of fluorescence and coefficients of variation of the positive bacterial populations as indicated elsewhere (Benedetti *et al*., 2016; Akkaya *et al*., 2018).

For the fluorimetry determinations, 200 μl of each diluted sample were distributed into a 96‐well microtiter plate (Corning & Costar, NY, USA) and incubated for 10 min at 30°C. After incubation, both the OD_600_ of the cell suspension and the fluorescence emission of PHP (λ_emission_ = 515 nm) were recorded at λ_excitation_ of either 405 nm or 485 nm with an *EnSpire*™ Plate Reader (Perkin‐Elmer, Waltham, MA, USA). In order to generate a calibration curve to calculate pH_i_, another set of cultures containing 10 ml of either LB medium or M9 minimal medium were inoculated with each bacterial strain. In the case of *P. putida* and *P. aeruginosa* strains, CCCP was added to the cultures at 20 μM (in the case of the standard curve for *E*. *coli,* the addition of CCCP was not necessary). The cultures were also grown to mid‐exponential phase as detailed before. One millilitre of each cell culture was spun down and resuspended into 1 ml of M9 medium salts containing 50 mM sodium benzoate and 50 mM methylamine HCl, and adjusted to the desired pH with 50 mM of the appropriate buffering agent [2‐(*N*‐morpholino)ethanesulfonic acid (MES) for pH = 5.5 to 6.5, 3‐(*N*‐morpholino)propanesulfonic acid (MOPS) for pH = 7.0 and 7.5, and *N*‐(Tris(hydroxymethyl)methyl)‐3‐aminopropanesulfonic acid (TAPS) for pH = 8.0 and 8.5]. The effect of adding sodium benzoate or methylamine on collapsing pH_i_ was evaluated with the fluorescent probe 5(6)‐carboxyfluorescein diacetate succinimidyl (see section below), and it was found that pH_i_ equalled the pH of the surrounding medium within a ± 0.5 units range. For both the PHP‐ and pHluorin2‐dependent determinations of pH_i_, culture samples (200 μl) were distributed into a 96‐well microtiter plate and both OD_600_ and fluorescence were also recorded as described in the preceding section. A Boltzmann sigmoid best‐fitting curve was applied to generate the calibration curves from the λ_excitation_ = 405 nm and λ_excitation_ = 485 nm excitation ratios (*R*_405/485_) obtained by incubating the cells into the buffered solutions, essentially as described by Martínez *et al*. (2012). The equation used to adjust the best‐fitting curve is as follows:

$$R\left(\mathrm{pH}\right)=\frac{R_{1}-R_{2}}{1+e^{\left(\mathrm{pH}-{\mathrm{pH}}_{0}\right)}/\varphi }+R_{2},$$

where *R*(pH) is the function representing the Boltzmann sigmoid best‐fitting curve; *R*_1_ and *R*_2_ are the lower and upper asymptotes of the curve (representing the minimum and maximum values of *R*_405/485_) respectively; pH is the actual pH_i_ value; *p*H_0_ is the half‐value for pH_i_ within the range assessed; and *φ* is the slope of the fitting curve. The pH_i_ values are expressed as the mean value of two measurements from at least three independent replicates ± SD.

In order to benchmark the PHP indicator against a well‐established protocol, the pH_i_ of the cells was calculated in some experiments by using the ratiometric fluorescent probe 5(6)‐carboxyfluorescein diacetate succinimidyl ester according to the standard protocols of Riondet *et al*. (1997) and Olivares Pacheco *et al*. (2017). Briefly, cells, grown as indicated in the text, were collected at an OD_600_ of 0.4–0.5 and centrifuged at 5000 *g* for 5 min at room temperature. The bacterial pellets were resuspended at a cell density of approximately 10^5^ cells ml^−1^ in 50 mM 4‐(2‐hydroxyethyl)‐1‐piperazineethanesulfonic acid (HEPES) buffer (pH = 9.0) containing 1 mM EDTA and incubated for 10 min at 30°C with 2.5 μM 5(6)‐carboxyfluorescein diacetate succinimidyl ester (Thermo Fisher Scientific, Waltham, MA, USA). Following this incubation, the samples were washed twice and resuspended in phosphate‐buffered saline buffer (pH = 7.0) containing 1 mM MgCl_2_. In order to eliminate any possible excess from the non‐conjugated probe, samples were treated with 10 mM glucose in phosphate‐buffered saline buffer. Cells were then centrifuged at 5000 *g* for 5 min, and bacterial pellets were washed twice and resuspended in a buffer composed of 50 mM Tris, 50 mM MES, 140 mM choline chloride, 1 mM MgCl_2_, 10 mM KCl, 10 mM NaHCO_3_ and 0.5 mM CaCl_2_ (pH = 7.0). One hundred fifty microlitres of each sample was placed on black 96‐well plates (Nunclon Δ Surface; Sigma‐Aldrich Co., St. Louis, MO, USA) and incubated at 30°C in a microtiter plate spectrophotometer (*Infinite 200 Pro*™; Tecan Trading AG, Männedorf, Switzerland). Fluorescence intensities were measured at excitation wavelengths λ_excitation_ = 490 nm (pH‐sensitive wavelength) and λ_excitation_ = 440 nm (pH‐insensitive wavelength). The emission wavelength used in all of these experiments was λ_emission_ = 525 nm. The excitation and emission intensities were used to calculate the corresponding ratios, which were associated to pH_i_ through a linear regression in a standard curve prepared with cells exposed to a set of calibrated buffers following the procedure described by Riondet *et al*. (1997). The pH_i_ values calculated with the chemical probe are expressed as the mean value of three measurements from at least three independent replicates ± SD.

### *In vivo* tracking of the dynamic changes of cytoplasmic pH upon pH shifts in the extracellular milieu

These experiments were carried out with 10 ml cultures of *E*. *coli* MG1655 or *P. putida* KT2440, each carrying plasmid pS2513·*PHP*, grown in M9 minimal medium added with 20 mM glucose from overnight pre‐cultures prepared as stated above. Once the cells reached mid‐exponential phase (OD_600_ = 0.4–0.5), 150‐μl aliquots of each culture were distributed into 96‐well microtiter plates and incubated for 10 min at 30 or 37°C (as appropriate for each strain) with orbital agitation at 180 rpm. The plate was transferred to the *EnSpire*™ Plate Reader, and the fluorescence emission was recorded continuously every 10 s as described previously. After 1 min incubation, 50 μl of acidic (pH = 5.5; buffered with 200 mM MES) or basic (pH = 8.5; buffered with 200 mM TAPS) M9 minimal medium containing 20 mM glucose was added to each well. Additionally, 50 μl of 250 mM sodium benzoate was added to a set of samples in order to fully collapse the pH_i_ of the cells as indicated above. The recording of the fluorescence emission was resumed immediately after these additions. The pH_i_ for each sample was calculated from the *R*_405/485_ values obtained for the fluorescence emission plotted against the standard curves prepared as described above.

## Conflict of interest

None declared.

## Supporting information


**Fig. S1.** Calibration curves for *E*. *coli* and *P*. *putida* carrying the PHP indicator in a complex culture medium.
**Fig. S2.** Calibration curve for *P*. *putida* carrying the PHP indicator in a minimal culture medium.
**Table S1.** Intracellular pH assessment of *P*. *putida* KT2440 and mutants in glycolytic genes in glucose cultures.
**Sequence S1.** Synthetic DNA encoding the PHP indicator.
