# Supplementary material for: Non‐invasive, ratiometric determination of intracellular pH in Pseudomonas species using a novel genetically encoded indicator
Source: Microb Biotechnol. 2019 Jun 4;12(4):799–813. doi: 10.1111/1751-7915.13439 (PMC6559197; doi:10.1111/1751-7915.13439)
Supplement: Supplementary file 1 — Fig. S1. Calibration curves for E. coli and P. putida carrying the PHP indicator in a complex culture medium. Fig. S2. Calibration curve for P. putida carrying the PHP indicator in a minimal culture medium. Table S1. Intracellular pH assessment of P. putida KT2440 and mutants in glycolytic genes in glucose cultures. Sequence S1. Synthetic DNA encoding the PHP indicator. [file MBT2-12-799-s001.pdf]

Supporting Information

Non-invasive, ratiometric determination of intracellular pH in *Pseudomonas* species using a novel genetically-encoded indicator

by

Alejandro Arce-Rodríguez, Daniel C. Volke, Sarina Bense,
Susanne Häussler, and Pablo I. Nikel

Sequence S1 · Synthetic DNA encoding the PHP indicator.

aagcttAGGAGGAAAAACAT**ATG**AGCAAAGGTGAAGAATTGTTCACCGGTGTGGTGCCA
ATTTTGGTCGAaCTCGATGGGGATGTGAACGGCCATAAGTTCTCGGTGAGCGGCGAAGG
GGAAGGGGATGCCACCTATGGCAAACTCACGCTCAAATTTATTTGCACCACCGGCAAGT
TGCCTGTCCCCTGGCCAACGCTGGTCACCACGTTGTCGTATGGTGTCCAATGCTTTTCC
CGGTATCCTGATCACATGAAACAACATGACTTTTTTAAAAGCGCGATGCCGGAAGGGTA
TGTGCAGGAGCGGACGATTTTTTTTAAGGACGACGGGAATTACAAGACGCGGGCAGAGG
TGAAGTTTGAAGGTGACACGTTGGTGAACCGTATTGAGTTGAAGGGTATCGACTTCAAG
GAAGATGGGAACATCCTCGGTCACAAACTGGAATATAATTACAACGAGCACCTCGTCTA
CATTATGGCGGACAAACAAAAAAATGGCACGAAAGCGATTTTCCAAGTCCACCATAACA
TTGAAGATGGGTCCGTCCAACTCGCCGACCATTACCAACAGAATACCCCAATCGGCGAT
GGGCCGGTGCTGCTCCCAGACAACCACTATCTCCATACGCAATCGGCGCTGTCCAAGGA
cCCGAATGAAAAACGTGATCATATGGTGCTGCTGGAGTTCGTCACCGCGGCTGGTATCA
CCCACGGTATGGATGAaCTCTACAAG**TAA**actagt

The individual *Hin*dIII and *Spe*I restriction targets are underlined at the beginning and by the end of the sequence, respectively. The coding sequence of the PHP indicator is highlighted in green, the *START* and *STOP* codons are indicated in boldface, and the synthetic ribosome binding site is shown in red.

Fig. S1 · Calibration curves for *E. coli* and *P. putida* carrying the PHP indicator in a complex culture medium.

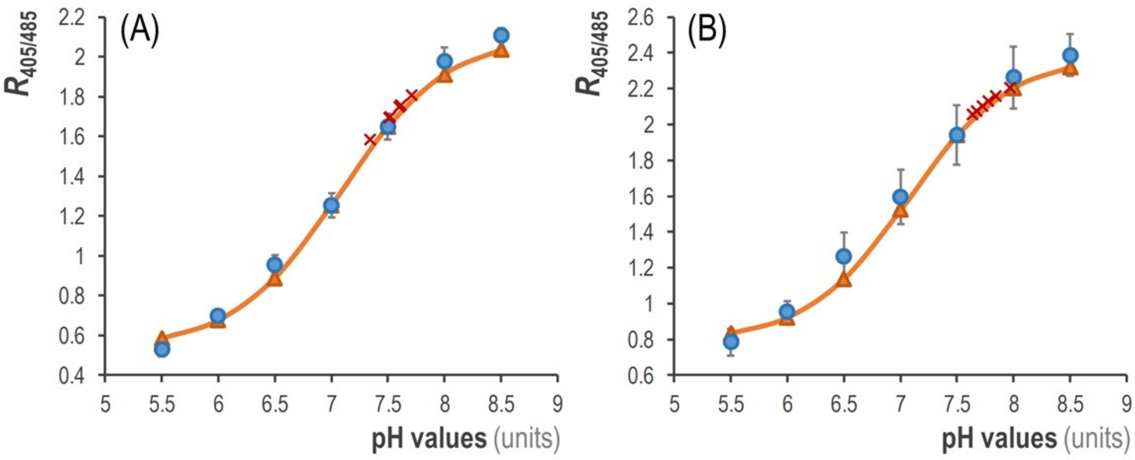

Separate calibration curves for intracellular pH values were calculated by means of the PHP-dependent fluorescence in *E. coli* DH5α (A) and *P. putida* KT2440 (B) transformed with plasmid pS2513·*PHP* and grown in LB medium at 30°C or 37°C, respectively. The data for the calibration curve was obtained as the ratio between the excitation peaks at $\lambda_{excitation}$ = 405 nm and 485 nm ($R_{405/485}$) plotted against the pH values of equilibrated cells (blue circles). These values were fitted using the Boltzmann sigmoid best-fitting curve to obtain the calibration curve (orange triangles), which was used to calculate the actual pH_i values in either strain during exponential growth (red crosses). Data points are representative of independent triplicates (mean values and standard deviation), and the red crosses (corresponding to six individual determinations), are shown in the curve to indicate the narrow dispersion of experimentally-calculated pH_i values.

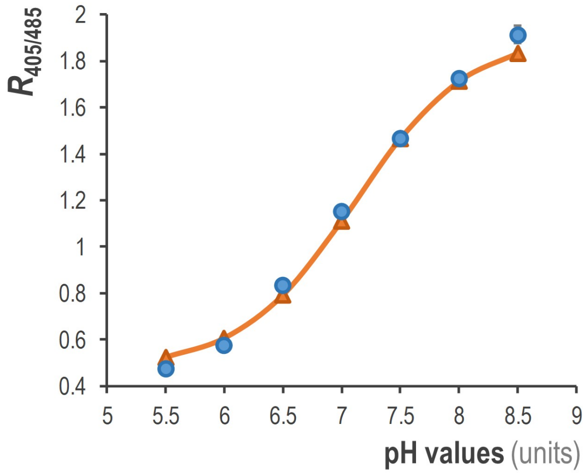

Fig. S2 · Calibration curve for *P. putida* carrying the PHP indicator in a minimal culture medium. A calibration curve for intracellular pH values was calculated by means of the PHP-dependent fluorescence in *P. putida* KT2440 carrying plasmid pS2513·*PHP* and grown in M9 minimal medium containing 20 mM glucose at 30°C. The data for the calibration curve was obtained as the ratio between the excitation peaks at $\lambda_{excitation}$ = 405 nm and 485 nm ($R_{405/485}$) plotted against the pH values of equilibrated cells (blue circles). These values were fitted using the Boltzmann sigmoid best-fitting curve to obtain the calibration curve (orange triangles), which was used to calculate the actual pH_i values in either strain during exponential growth. Data points are representative of independent triplicates (mean values and standard deviation).

Table S1. Intracellular pH assessment of *P. putida* KT2440 and mutants in glycolytic genes in glucose cultures.

**Bacterial strain**	**pH values during**			
	**Exponential phase**		**Stationary phase**	
	**Cytoplasmic**	**Extracellular**	**Cytoplasmic**	**Extracellular**
P. putida KT2440	7.66 ± 0.09	6.35 ± 0.01	8.01 ± 0.12	6.84 ± 0.01
P. putida Δ*glk*	7.72 ± 0.09	6.05 ± 0.01	8.09 ± 0.14	6.45 ± 0.01
P. putida Δ*gcd*	7.59 ± 0.06	7.03 ± 0.01	7.63 ± 0.04	7.04 ± 0.01

All strains, carrying plasmid pS2513·*PHP*, were grown in M9 minimal medium containing 20 mM glucose, and the cytoplasmic pH was determined both during exponential growth and in the stationary phase (i.e. 24 h post-inoculation of the cultures). The pH of the culture medium was likewise measured in culture supernatants from the same samples used for cytoplasmic pH determinations. These results represent the mean value of each parameter ± standard deviation of triplicate measurements from at least three independent experiments.
